# Novel influenza A(H1N2) seasonal reassortant identified in a patient sample, Sweden, January 2019

**DOI:** 10.2807/1560-7917.ES.2019.24.9.1900124

**Published:** 2019-02-28

**Authors:** Åsa Wiman, Theresa Enkirch, AnnaSara Carnahan, Blenda Böttiger, Tove Samuelsson Hagey, Per Hagstam, Rosmarie Fält, Mia Brytting

**Affiliations:** 1 Unit for Laboratory Surveillance of Viral Pathogens and Vaccine Preventable Diseases, Department of Microbiology, the Public Health Agency of Sweden, Solna, Sweden; 2 Authors contributed equally to the work and share first authorship; 3 Unit for Vaccination Programmes, Department of Communicable Disease Control and Health Protection, the Public Health Agency of Sweden, Solna, Sweden; 4 Clinical Microbiology Laboratory, Lund, Sweden; 5 Regional Office of Communicable Disease Control and Prevention, Region Skåne, Malmö, Sweden

**Keywords:** human influenza, reassortant viruses, whole genome sequencing, haemagglutinin, neuraminidase, surveillance, Sweden

## Abstract

In January 2019, a human seasonal reassortant influenza A(H1N2) virus with a novel 7:1 genetic constellation was identified in a 68-year-old female patient with suspected pneumonia. The virus harboured A(H3N2) neuraminidase and remaining genes from A(H1N1)pdm09. The patient recovered after severe illness. No additional cases have been detected. This is the second identified A(H1N2) seasonal reassortant in a human in Europe within 1 year; a previous case was detected in the Netherlands in March 2018.

As part of Swedish national influenza surveillance, a seasonal reassortant influenza A(H1N2) virus with a novel genetic constellation was identified. This is the second detected seasonal A(H1N2) reassortant in a human in Europe within 1 year. Here, we describe the detection of the virus, its genetic characteristics and follow-up investigations.

## Influenza surveillance in Sweden

In Sweden, surveillance of influenza is conducted at the Public Health Agency of Sweden (PHAS), which acts as the National Influenza Centre. Samples included in national virological surveillance are referred to PHAS from either sentinel general practitioners (nasal swabs from cases with influenza-like illness, (ILI) or diagnostic laboratories (influenza virus-positive samples). Two of these laboratories (in Lund and Gothenburg, respectively) perform influenza virus subtyping. At PHAS, the viruses are typed and sub/lineage-typed by real-time PCR and a subset of the influenza virus-positive samples are analysed by whole genome sequencing (WGS), isolated on cell culture and further analysed for sensitivity to neuraminidase (NA) inhibitors by NA inhibition assay.

## Influenza season 2018/19 as at week 5 2019

According to national surveillance, the influenza epidemic in Sweden began in week 50 2018. Over the following 2 weeks, the number of laboratory-confirmed influenza cases increased, indicating medium intensity at week 52 before decreasing to a low level of intensity until week 3 2019. Influenza activity then increased again reaching medium intensity in week 5. The 2018/19 influenza season is expected to reach its peak in February 2019.

**Figure 1:**
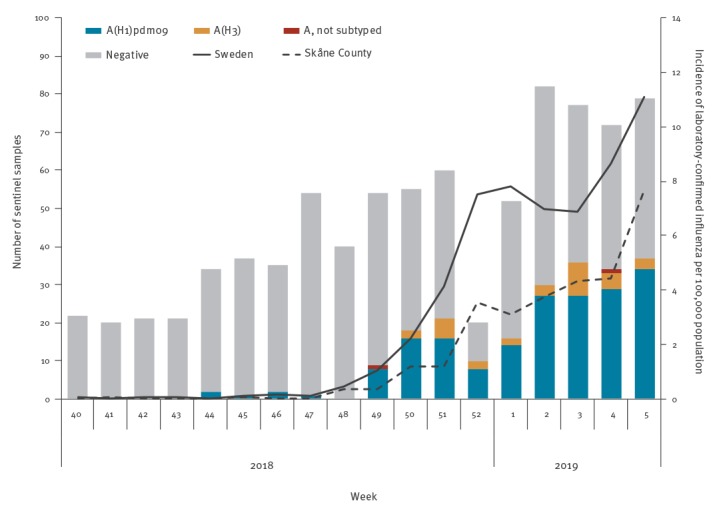
Weekly number of positive influenza A virus samples, by subtype from sentinel surveillance and incidence of laboratory-confirmed influenza A in Sweden and Skåne County, week 40 2018–week 5 2019

So far, the 2018/19 influenza season (week 40 2018–week 5 2019) has been dominated by influenza A virus (> 99% and 98% in laboratory and sentinel surveillance, respectively). As at week 5, a cumulative total of 5,899 laboratory-confirmed influenza cases had been reported from the regional diagnostic laboratories in Sweden including in Skåne where the A(H1N2) virus was detected. Of the cases, 5,867 were positive for influenza A virus and 32 for influenza B virus. In total, 611 of these influenza A-virus-positive samples were subtyped and 80% were influenza A(H1)pdm09 virus and 20% were influenza A(H3) virus. In addition, from week 40 through week 5, 838 sentinel samples were submitted to PHAS of which, 220 tested positive for influenza A virus; 187 (85%) of influenza A-virus-positive samples were influenza A(H1N1)pdm09 virus and 33 (15%) were influenza A(H3N2) virus. (Figure 1).

The four positive influenza B sentinel samples are not included in the figure.

All 72 influenza A(H1)pdm09 viruses, for which the haemagglutinin (HA) gene has been sequenced so far this season, belong to genetic group 6B.1. The dominating genetic group among the characterised influenza A(H3) viruses is 3C.2a1b (n = 19); 3C.2a2 (n = 5) and 3C.3a (n = 2) were also detected.

## Case description

In the last week of December (week 52 2018), a 68-year-old female patient with a history of chronic obstructive pulmonary disease consulted her primary care physician following 5 days of fever up to 40°C. On clinical suspicion of pneumonia, she was referred to a local hospital in Skåne County where she was hospitalised. A nasopharyngeal swab taken on the day of admission was positive for influenza A virus and the patient was treated with oseltamivir (75 mg, two times daily) for 5 days. She recovered quickly and was discharged 5 days after admission. The patient had not been vaccinated against influenza during the 2018/19 season.

Diagnosis of influenza A virus infection at the local hospital was performed by real-time PCR using Simplexa Flu A/B and RSV direct kit, (DiaSorin Molecular LLC, California, United States (US)). The sample was forwarded to the Clinical Microbiology Laboratory in Lund for subtyping (as are all influenza A virus-positive samples in Skåne County) with in-house real-time PCRs targeting H3 and N1pdm09 [1]. As this sample was negative in these assays, it was forwarded to the PHAS where influenza A(H1)pdm09 virus was detected by in-house real-time PCR. The presence of A(H1)pdm09 virus was also subsequently confirmed by Filmarray Respiratory Panel BioFire (Diagnostics LLC, Utah, US) at the Clinical Microbiology Laboratory in Lund.

## Genetic characterisation

The virus, initially subtyped as A(H1)pdm09 by real-time PCR, was revealed as A(H1N2) on 22 January 2019, after WGS on an Ion Torrent platform (Thermo Fisher Scientific, Waltham, Massachusetts, US). Seven segments (HA, matrix (M) non-structural (NS), polymerase components PB1, PB2 and PA and nucleoprotein (NP)) of this virus are derived from seasonal A(H1N1)pdm09 virus, while the neuraminidase (NA) segment is derived from seasonal A(H3N2) virus. The gene sequence of this strain, A/Ystad/1/2018, is available from the Global Initiative on Sharing All Influenza Data (GISAID) EpiFlu database (EPI_ISL_336041) [2,3].

Phylogenetic analysis was conducted using sequences of influenza viruses recently circulating in Sweden and reference datasets provided by the World Health Organization Collaborating Centre (WHO CC) in London through the European Centre for Disease Prevention and Control for the 2018/19 season.

**Figure 2:**
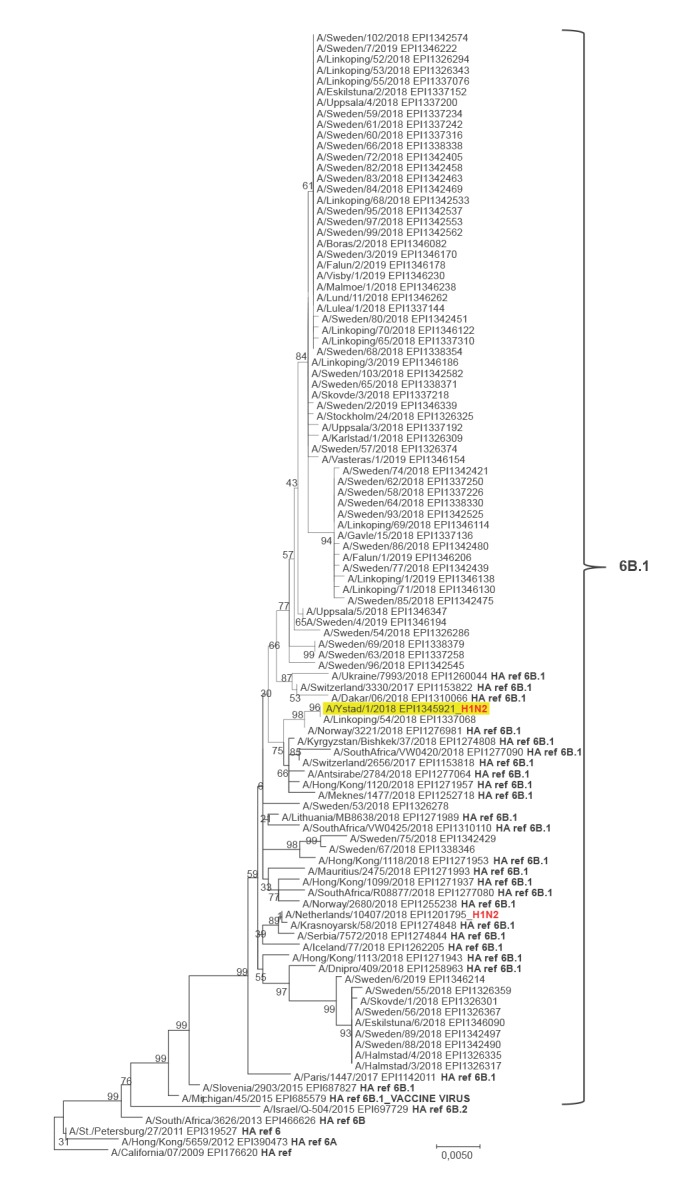
Phylogenetic analysis of influenza A(H1N2) virus A/Ystad/1/2018 haemagglutinin (HA) genome segment^a^

^a^ Influenza A(H1N2) virus A/Ystad/1/2018 haemagglutinin (HA) genome segment is indicated in yellow.

 The HA gene segment clusters within genetic group 6B.1 (Figure 2) with the nucleotide (nt) sequence being identical to the recent Swedish virus A/Linkoping/54/2018 (collected 28 November 2018), except in one nt position where A/Linkoping/54/2018 had a silent mutation (ambiguous nt). Compared with A/California/07/2009 (GISAID EPI176620), A/Ystad/1/2018 and A/Linkoping/54/2018 have the HA1 amino acid substitutions S74, P83S, S84N, D97N, T120A, S162N, K163Q, S164T, S183P, S185T, S190I, S203T, I216T, R223Q, A256T, K283E, I295V, I321V and the HA2 substitutions E47K, S124N and E172K. Thus, with exception of HA1 S190I, they are identical to the reference viruses A/Norway/3221/2018 (EPI1276981) and A/Lithuania/MB8638/2018 (EPI1271989).

Phylogenetic analysis is in the context of recent Swedish influenza A (H1N1)pdm09 viruses, the influenza A(H1N2) reassortant detected in the Netherlands [15] and a reference influenza virus dataset.

Collection dates of the Swedish viruses range from 31 October to 10 January 2019. Reference datasets were provided by the World Health Organization Collaborating Centre London through the European Centre for Disease Prevention and Control for seasonal (2018/19) phylogenetic analysis.

Phylogenetic trees were constructed by the maximum likelihood method in MEGA7 [19]. Bootstrap values displayed on branches are based on 500 replicates.

**Figure 3:**
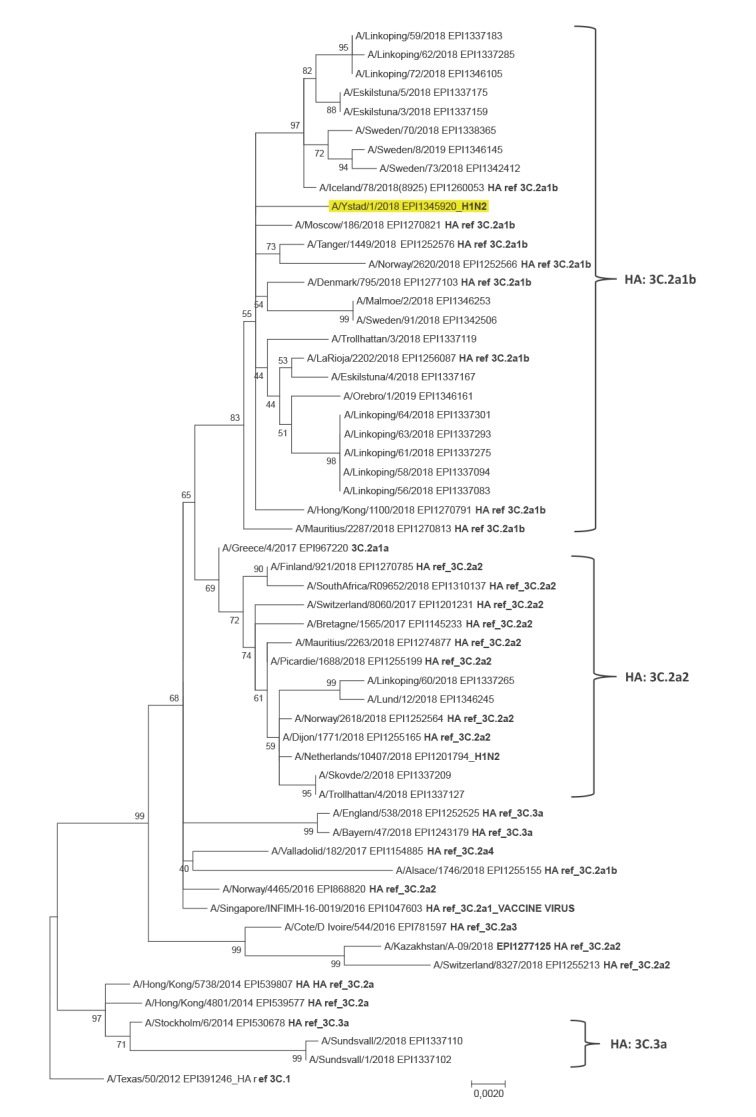
Phylogenetic analysis of the influenza A(H1N2) virus A/Ystad/1/2018 neuraminidase (NA) genome segment^a^

^a^ Influenza A(H1N2) virus A/Ystad/1/2018 neuraminidase (NA) genome segment is indicated in yellow.

In the phylogenetic analysis of the NA sequence (Figure 3), A/Ystad/1/2018 clusters with NA sequences of the A(H3N2)-viruses that belong to the HA genetic group 3C.2a1b. Compared with recent Swedish A(H3N2)-viruses, the NA nt sequence of A/Ystad/1/2018 is unique. On the amino acid level, A/Ystad/1/2018 is identical to the reference virus A/Tanger/1449/2018. All amino acid substitutions in comparison to A/Texas/50/2012 (EPI391246 in GISAID) namely P126L, H150R, K220N, E221D, S245N, S247T, T267K, V303I, N329S, D339N, I380V and P468H are also present in 18 of 24 recent Swedish viruses included in the analysis, but all of them have one or more additional substitutions. A/Ystad/1/2018 does not harbour any of the NA mutations that are known to be associated with reduced or highly reduced inhibition to NA inhibitors.

Phylogenetic analysis is in the context of recent Swedish influenza A(H3N2) viruses, the influenza A(H1N2) reassortant detected in the Netherlands [15] and a reference influenza virus dataset.

Collection dates of the Swedish viruses range from 1 December to 4 January 2019. Reference datasets were provided by the World Health Organization Collaborating Centre London through the European Centre for Disease Prevention and Control for seasonal (2018/19) phylogenetic analysis.

Phylogenetic trees were constructed by the maximum likelihood method in MEGA7 [19]. Bootstrap values displayed on branches are based on 500 replicates.

In the phylogenetic analysis of the PB2, PB1, PA, NP, M and NS gene segments (in the context of recent Swedish A(H1N1)pdm09 viruses), A/Ystad/1/2018 is located on the same branch as the recent Swedish A(H1N1)pdm09 virus A/Linkoping/54/2018 (Supplement S1). For the M and NS segments, two additional recent Swedish viruses (for which only M and NS (and for one case the NA) have been sequenced) share the same branch. The M2 harboured the amino acid substitution S31N, which causes resistance to amantadine. The virus was isolated on cell culture and sent along with the original sample to the WHO CC in London for further analysis.

## Follow-up measures

Onset of symptoms occurred on the same day that the patient returned home to Skåne County (the southern most county of Sweden) from a 4-day visit to a relative living in Stockholm County who had symptoms of a respiratory infection; the relative had no history of recent travel.

As at 24 February 2019 no additional human seasonal A(H1N2) reassortant viruses have been detected in Sweden. In Skåne County, 520 influenza A virus-positive samples that were collected after the patient returned home have been subtyped to either H3 or N1pdm09 indicating that the reassortant virus did not spread in the county.

In addition, 32 A(H1)pdm09-positive samples collected week 1–5 2019, including 10 sentinel samples from Skåne County, have been determined as A(H1N1)pdm09 by WGS or real-time PCR at the PHAS – this was also the case for the 63 A(H1)pdm09-positive samples collected up to week 52 2018 throughout Sweden. The detection of the A(H1N2) reassortant virus was reported to the WHO CC London on 23 January, announced on the public website of the PHAS on 24 January and reported via the European early warning reporting system (EWRS) on 25 January and according to international health regulations (IHR) to WHO on 30 January.

## Discussion

While co-infections with seasonal A(H1N1) and A(H3N2) influenza strains are not unusual [4-7], only a few studies have described reassortant viruses as a consequence of such co-infections [8-10]. This suggests that reassortment is a rare occurrence and that reassorted A(H1N2)-viruses do not easily spread between humans [11], with the exception of A(H1N2) reassortant viruses circulating in 1988/89 in China [12,13] and worldwide between 2001 and 2003 [14]. Interestingly, a human natural infection with an A(H1N2) reassortant virus harbouring gene segments from seasonal influenza A(H1N1)pdm09 virus (HA and NS) and A(H3N2) virus (PB2, PB1, PA, NP, NA and M) was described as recently as March 2018, by Meijer et al. [15]. In accordance with the hypothesis of limited spread, no further cases were observed, as is the case also (as at 24 February 2019) for the Swedish A(H1N2) reassortant.

Real-time PCR assays targeting at least two genes can detect or give an indication of a reassortment, depending on the combination of targets used and segments reassorted, respectively. Targeting both the HA and NA genes is of interest because a change of the HA and NA constellation might have a possible impact on immune protection. However, WGS provides a powerful tool to both detect and characterise reassortants of all eight gene segments. Here, WGS showed that A/Ystad/1/2018 harbours seven gene segments from seasonal A(H1N1)pdm09 virus and one segment (NA) from A(H3N2) virus.

To our knowledge, this is the first human seasonal A(H1N2) reassortant with this gene segment constellation detected in humans. Since the HA is closely related genetically to that of the A(H1N1)pdm09 viruses circulating in Sweden so far during the 2018/19 season, we expect no difference in vaccine effectiveness (VE) against this reassortant virus compared with seasonal A(H1N1)pdm09 viruses. This season, substantial interim VE has been shown against circulating A(H1N1)pdm09 viruses in studies from Canada, Hong Kong and Europe [16-18]. Antigenic characterisation of this reassortant virus will be conducted at WHO CC.

In conclusion, our results support the observation that the currently co-circulating viruses of A(H1N1)pdm09 and seasonal A(H3N2) viruses have the potential to reassort and form new strains that can spread globally causing epidemics. Reassortment between seasonal and zoonotic influenza might lead to novel pandemic strains and therefore molecular surveillance of circulating influenza strains is of high importance.

## Supplementary Data

376000 Supplement S1

## Supplementary Data

41000 Supplement S2
