# Supplementary material for: Novel influenza A(H1N2) seasonal reassortant identified in a patient sample, Sweden, January 2019
Source: Euro Surveill. 2019 Feb 28;24(9):1900124. doi: 10.2807/1560-7917.ES.2019.24.9.1900124 (PMC6402178; doi:10.2807/1560-7917.ES.2019.24.9.1900124)

Supplement 1:
Phylogenetic analysis of M, NS, PB1, PB2, NP and PA genome segments of the seasonal reassortant virus A/Ystad/1/2018.

This supplementary material is hosted by Eurosurveillance as supporting information alongside the article "Novel influenza A(H1N2) seasonal reassortant identified in a patient sample, Sweden, January 2019" on behalf of the authors who remain responsible for the accuracy and appropriateness of the content. The same standards for ethics, copyright, attributions and permissions as for the article apply. Eurosurveillance is not responsible for the maintenance of any links or email addresses provided therein.

Phylogenetic analysis of the H1N2 reassortant A/Ystad/1/2018 (in yellow) in the context of recent Swedish A(H1N1)pdm09 viruses (collection dates 31 October to 10 January 2019) and the vaccine virus for season 2018/19 A/Michigan/45/2015: In the analysis of NS the H1N2 reassortant detected in the Netherlands in March 2018 [1] is included. For eleven of the Swedish viruses included in the M and NS1 analyses, the NA subtype remains unconfirmed due to incomplete sequencing results (where successful sequencing were only obtained for shorter fragments). The PB2, PB1, PA, NP, M and NS genome segments A/Ystad/1/2018 are located on the same branch as A/Linkoping/54/2018. For the M and NS segments two additional recent Swedish viruses (with incomplete sequencing results for the other gene segments) share the same branch. Reference dataset for seasonal (2018/2019) phylogenetic analysis were provided by the World Health Organization Collaborating Centre London through the European Centre for Disease Prevention and Control (ECDC). We gratefully acknowledge the authors, originating and submitting laboratories of the sequences retrieved from GISAID's Epiflu database that were used in this analysis (Supplement 2). Phylogenetic trees were constructed using the maximum likelihood method in MEGA7 [2] software. Bootstrap values displayed on branches are based on 500 replicates.

1. Meijer, A., et al., Case of seasonal reassortant A(H1N2) influenza virus infection, the Netherlands, March 2018. Euro Surveill, 2018. **23**(15).

2. Kumar, S., G. Stecher, and K. Tamura, MEGA7: Molecular Evolutionary Genetics Analysis Version 7.0 for Bigger Datasets. Mol Biol Evol, 2016. **33**(7): p. 1870-4.

A (M; A(H1N1)pdm09 origin)

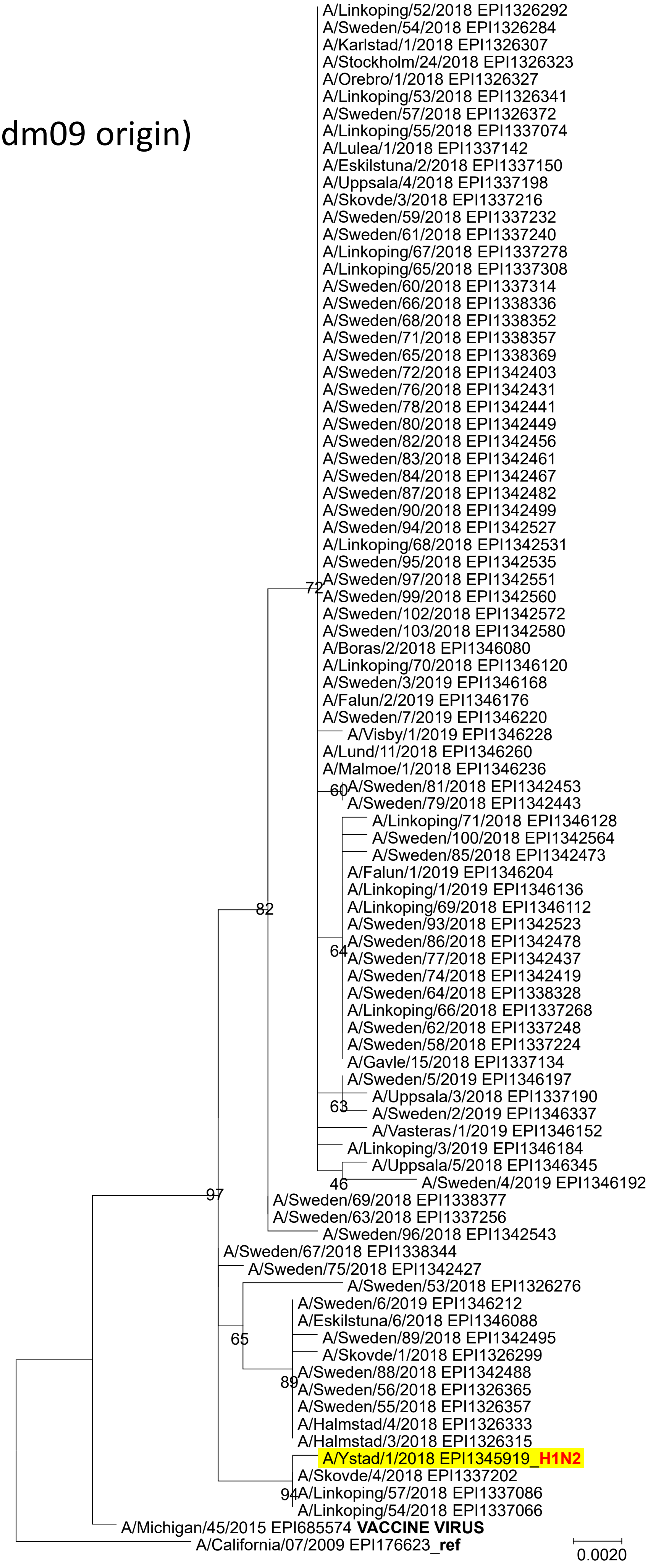

B (NS; A(H1N1)pdm09 origin)

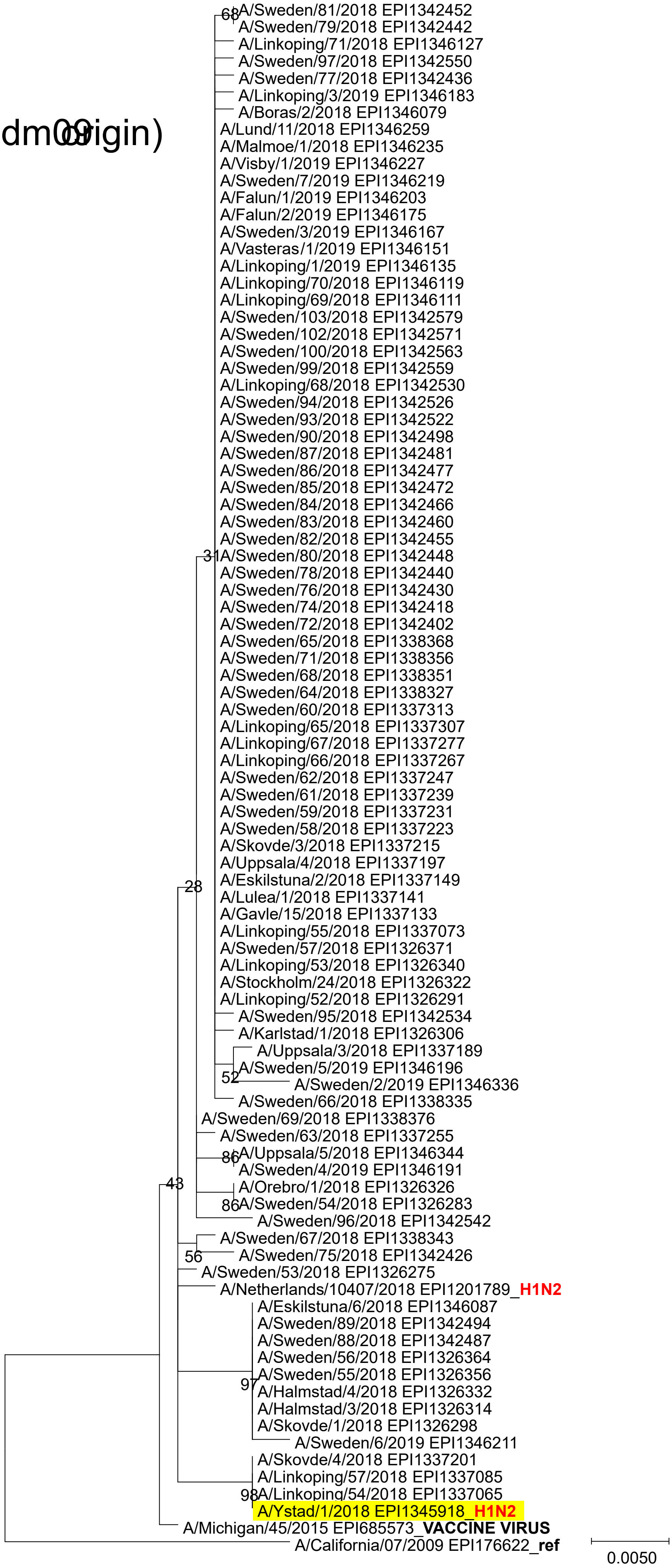

C (PB1; A(H1N1)pdm09 origin)

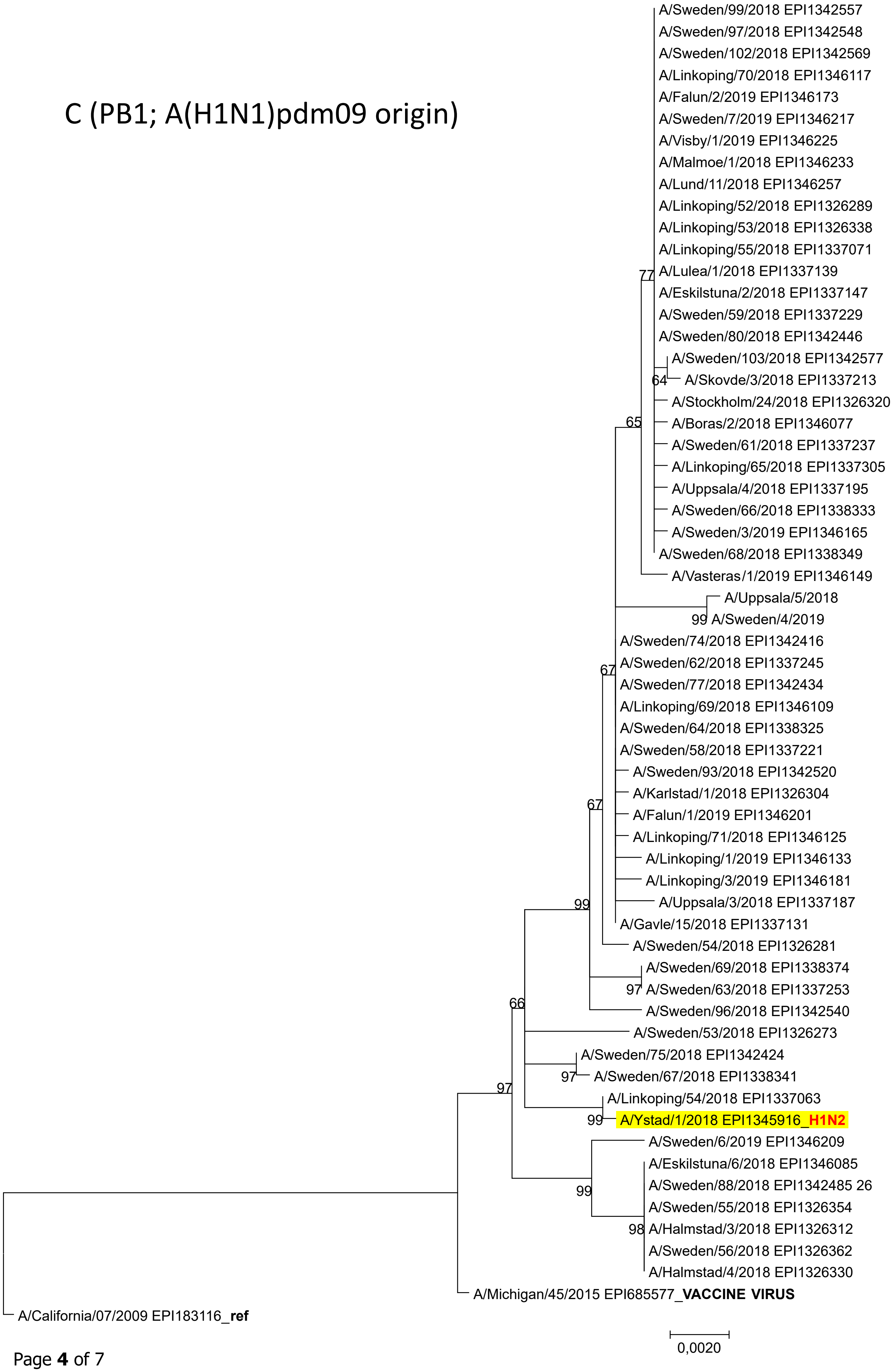

D (PB2; A(H1N1)pdm09 origin)

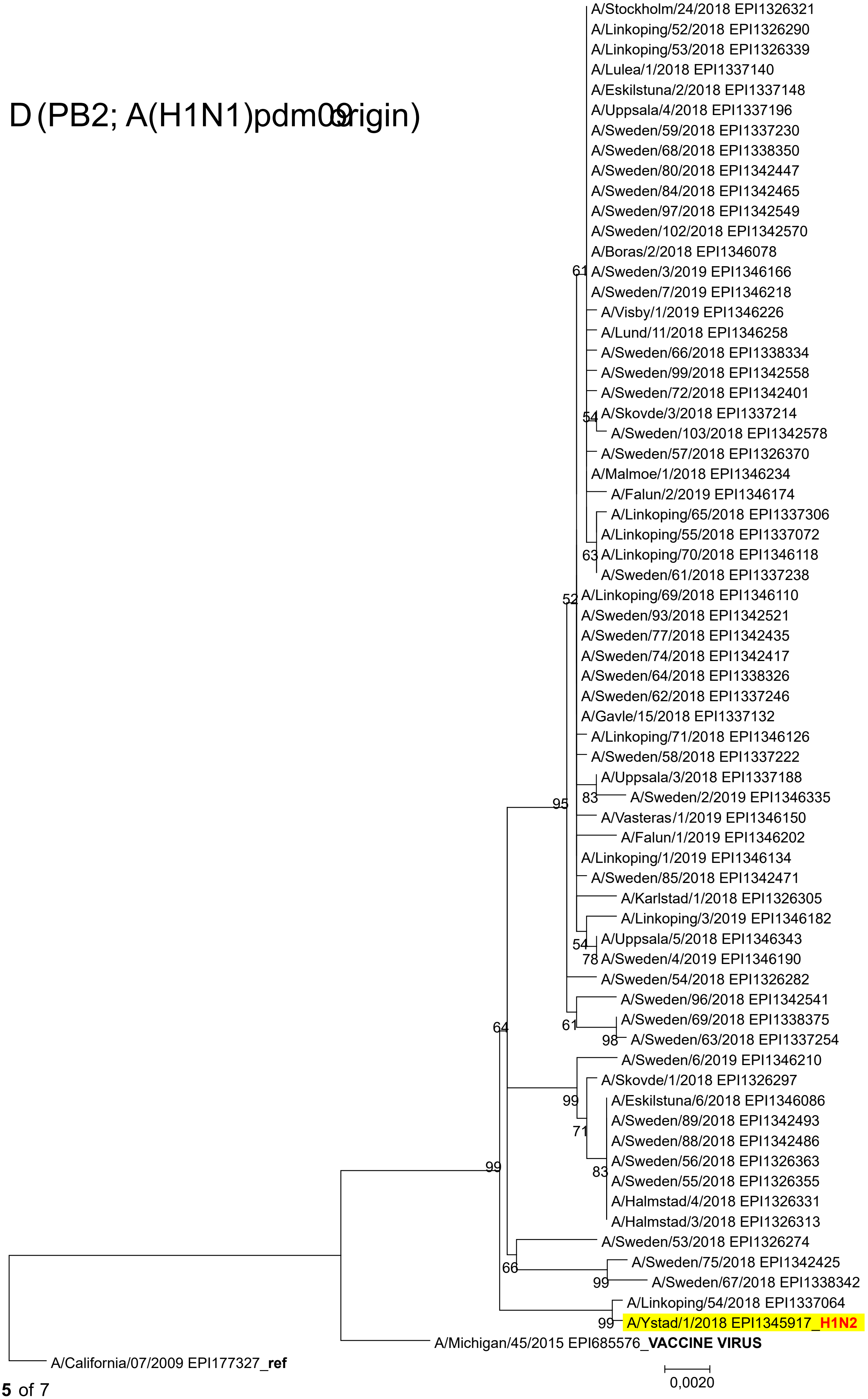

E (NP; A(H1N1)pdm09 origin)

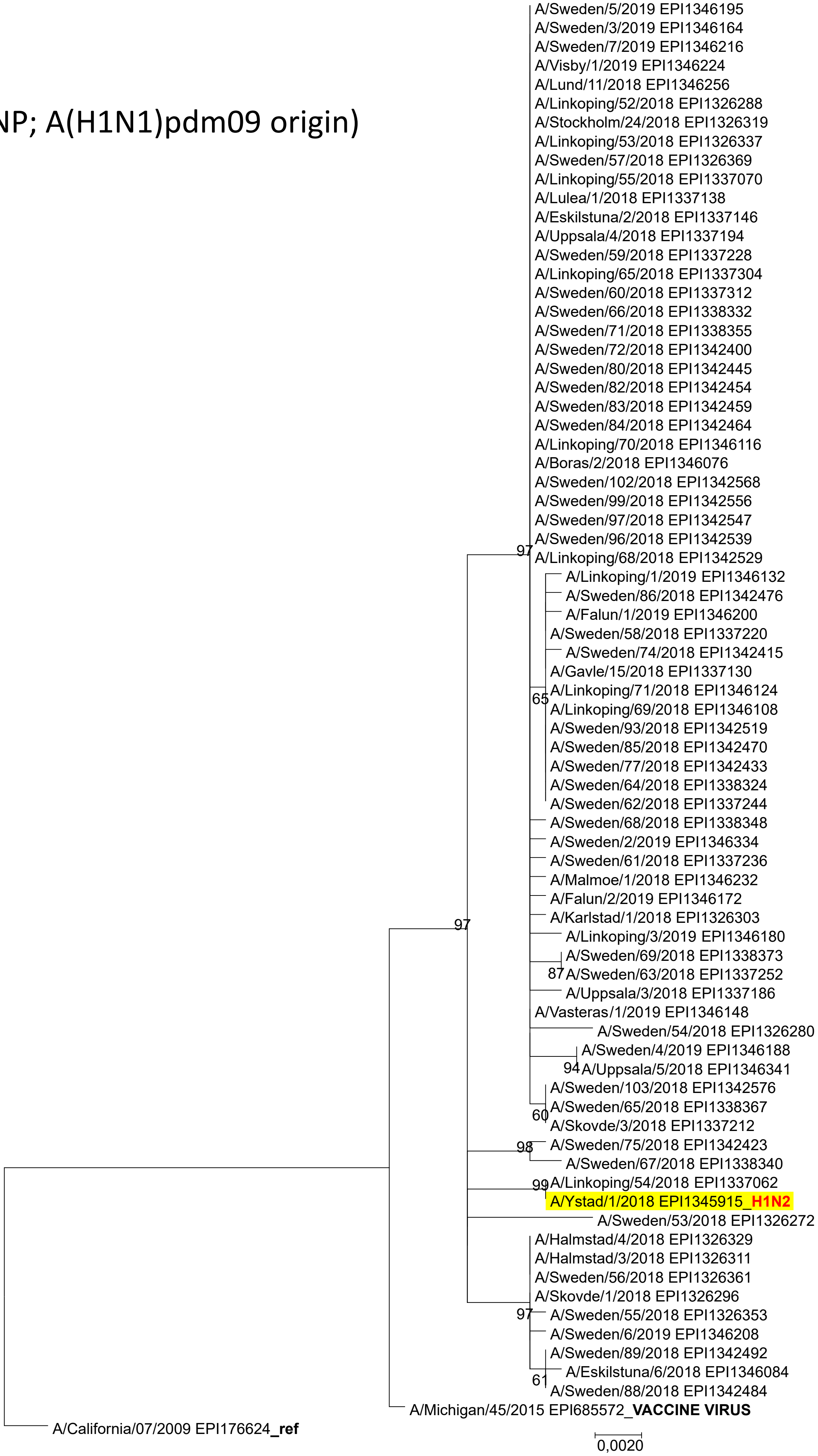

B (PA; A(H1N1)pdm09 origin)

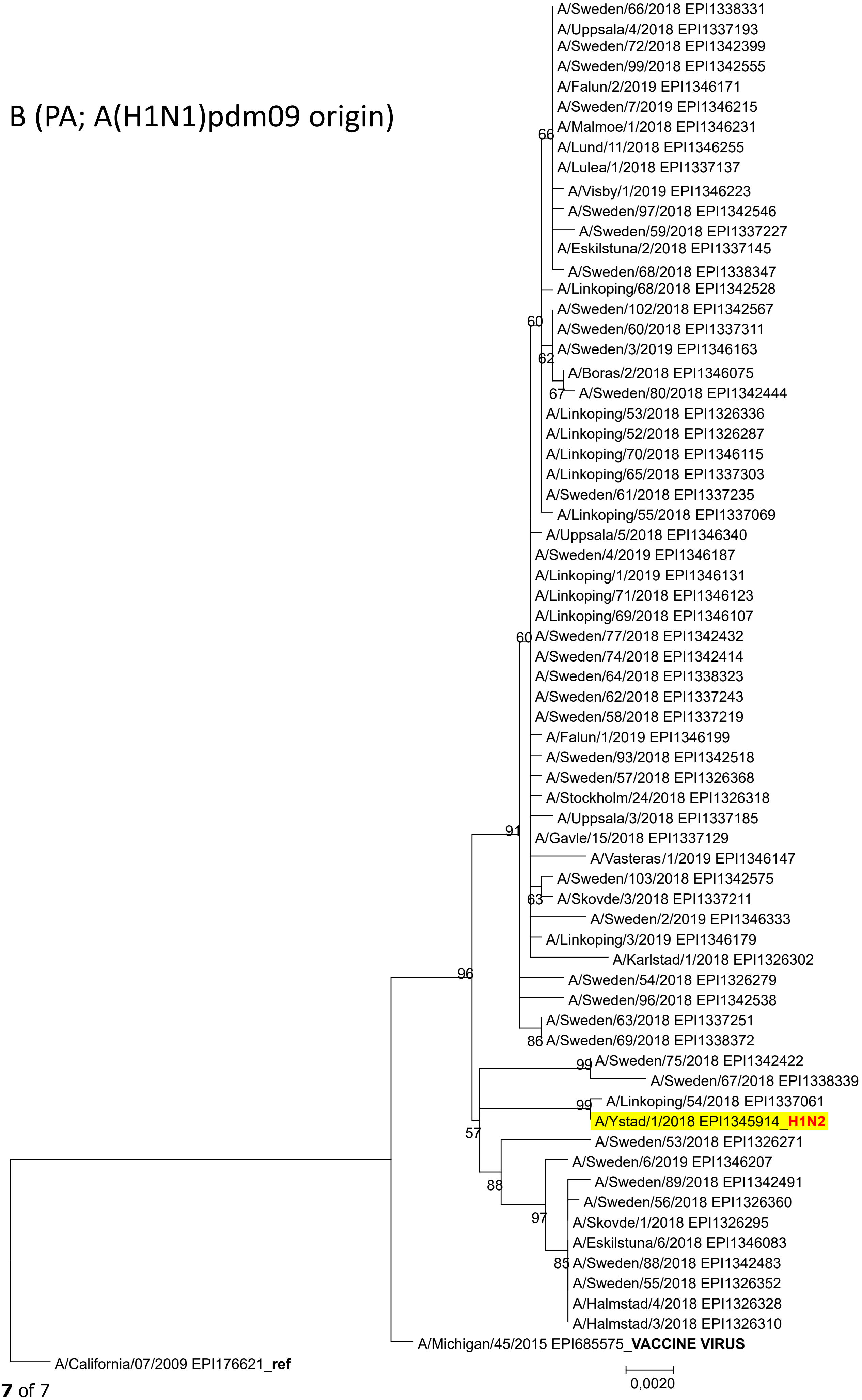

Supplement: Supplement S1 [file 1900124_ENKIRCH_SupplementS1.pdf]
