# Supplementary material for: Novel influenza A(H1N2) seasonal reassortant identified in a patient sample, Sweden, January 2019
Source: Euro Surveill. 2019 Feb 28;24(9):1900124. doi: 10.2807/1560-7917.ES.2019.24.9.1900124 (PMC6402178; doi:10.2807/1560-7917.ES.2019.24.9.1900124)
Supplement: Supplement S2 [file 1900124_ENKIRCH_SupplementS2.docx]

**Supplement 2. GISAID Acknowledgement tables.**

This supplementary material is hosted by *Eurosurveillance* as supporting information alongside the article “Novel influenza A(H1N2) seasonal reassortant identified in a patient sample, Sweden, January 2019” on behalf of the authors who remain responsible for the accuracy and appropriateness of the content. The same standards for ethics, copyright, attributions and permissions as for the article apply. *Eurosurveillance* is not responsible for the maintenance of any links or email addresses provided therein.

We acknowledge the authors, originating and submitting laboratories of the sequences from GISAID’s EpiFlu ™ Database on which this research is based. All submitters of data may be contacted directly via the GISAID website [www.gisaid.org](http://www.gisaid.org).

Table 1. Seasonal A(H1N2) reassortant Netherlands, March 2018.

| **Segment ID** | **Segment** | **Isolate Name** | **Collection Date** | **Country** | **Submitting_Lab** | **Authors** | **Originating_Lab** |
| --- | --- | --- | --- | --- | --- | --- | --- |
| EPI1201795 | HA | A/Netherlands/10407/2018 | 05 Mar 2018 | Netherlands | Erasmus Medical Center | Meijer, A.; van den Brink, S.; Bestebroer, T.; Fouchier, R. | National Institute for Public Health and the Environment (RIVM) |
| EPI1201794 | NA | A/Netherlands/10407/2018 | 05 Mar 2018 | Netherlands | Erasmus Medical Center | Meijer, A.; van den Brink, S.; Bestebroer, T.; Fouchier, R. | National Institute for Public Health and the Environment (RIVM) |
| EPI1201789 | NS | A/Netherlands/10407/2018 | 05 Mar 2018 | Netherlands | Erasmus Medical Center | Meijer, A.; van den Brink, S.; Bestebroer, T.; Fouchier, R. | National Institute for Public Health and the Environment (RIVM) |

Table 2. Influenza A(H3N2) viruses WHO Collaboration Centre reference set for 2018/19 season.

| **Segment ID** | **Segment** | **Isolate Name** | **Collection Date** | **Country** | **Submitting_Lab** | **Authors** | **Originating_Lab** |
| --- | --- | --- | --- | --- | --- | --- | --- |
| EPI1142011 | HA | A/Paris/1447/2017 | 20 Oct 2017 | France | Crick Worldwide Influenza Centre |  | Institut Pasteur |
| EPI697729 | HA | A/Israel/Q-504/2015 | 15 Dec 2015 | Israel | Crick Worldwide Influenza Centre |  | Central Virology Laboratory Israel (NIC) |
| EPI1255238 | HA | A/Norway/2680/2018 | 16 Apr 2018 | Norway | Crick Worldwide Influenza Centre |  | WHO National Influenza Centre |
| EPI1271993 | HA | A/Mauritius/2475/2018 | 04 Jul 2018 | Mauritius | Crick Worldwide Influenza Centre |  | Central Health Laboratory |
| EPI1277064 | HA | A/Antsirabe/2784/2018 | 18 Jun 2018 | Madagascar | Crick Worldwide Influenza Centre |  | Institut Pasteur de Madagascar |
| EPI1271957 | HA | A/Hong Kong/1120/2018 | 18 Jun 2018 | Hong Kong (SAR) | Crick Worldwide Influenza Centre |  | Government Virus Unit |
| EPI1271953 | HA | A/Hong Kong/1118/2018 | 17 Jun 2018 | Hong Kong (SAR) | Crick Worldwide Influenza Centre |  | Government Virus Unit |
| EPI1271943 | HA | A/Hong Kong/1113/2018 | 15 Jun 2018 | Hong Kong (SAR) | Crick Worldwide Influenza Centre |  | Government Virus Unit |
| EPI1271937 | HA | A/Hong Kong/1099/2018 | 13 Jun 2018 | Hong Kong (SAR) | Crick Worldwide Influenza Centre |  | Government Virus Unit |
| EPI1274844 | HA | A/Serbia/7572/2018 | 03 Apr 2018 | Serbia | Crick Worldwide Influenza Centre |  | Institute of Immunology and Virology Torlak |
| EPI1258963 | HA | A/Dnipro/409/2018 | 03 Apr 2018 | Ukraine | Crick Worldwide Influenza Centre |  | Institute of Epidemiology and Infectious Diseases AMS of Ukraine |
| EPI1310110 | HA | A/SouthAfrica/VW0425/2018 | 11 Jun 2018 | South Africa | Crick Worldwide Influenza Centre |  | Sandringham, National Institute for Communicable D |
| EPI1277090 | HA | A/SouthAfrica/VW0420/2018 | 13 Jun 2018 | South Africa | Crick Worldwide Influenza Centre |  | Sandringham, National Institute for Communicable D |
| EPI1277080 | HA | A/SouthAfrica/R08877/2018 | 14 Jun 2018 | South Africa | Crick Worldwide Influenza Centre |  | Sandringham, National Institute for Communicable D |
| EPI1252718 | HA | A/Meknes/1477/2018 | 15 Mar 2018 | Morocco | Crick Worldwide Influenza Centre |  | Institut National d'Hygi&egrave;ne |
| EPI687827 | HA | A/Slovenia/2903/2015 | 26 Oct 2015 | Slovenia | Crick Worldwide Influenza Centre |  | Laboratory for Virology, National Institute of Public Health |
| EPI1262205 | HA | A/Iceland/77/2018 | 12 Apr 2018 | Iceland | Crick Worldwide Influenza Centre |  | Landspitali - University Hospital |
| EPI1271989 | HA | A/Lithuania/MB8638/2018 | 14 Mar 2018 | Lithuania | Crick Worldwide Influenza Centre |  | Lithuanian AIDS Center Laboratory |
| EPI1310066 | HA | A/Dakar/06/2018 | 26 Jun 2018 | Senegal | Crick Worldwide Influenza Centre |  | Institut Pasteur de Dakar |
| EPI1153822 | HA | A/Switzerland/3330/2017 | 20 Dec 2017 | Switzerland | Crick Worldwide Influenza Centre |  | Hopital Cantonal Universitaire de Geneves |
| EPI1153818 | HA | A/Switzerland/2656/2017 | 21 Dec 2017 | Switzerland | Crick Worldwide Influenza Centre |  | Hopital Cantonal Universitaire de Geneves |
| EPI1260044 | HA | A/Ukraine/7993/2018 | 04 Apr 2018 | Ukraine | Crick Worldwide Influenza Centre |  | Ministry of Health of Ukraine |
| EPI1274848 | HA | A/Krasnoyarsk/58/2018 | 03 May 2018 | Russian Federation | Crick Worldwide Influenza Centre |  | State Research Center of Virology and Biotechnology (VECTOR) |
| EPI1274808 | HA | A/Kyrgyzstan Bishkek/37/2018 | 10 Apr 2018 | Kyrgyzstan | Crick Worldwide Influenza Centre |  | National Virology Laboratory, Center Microbiological Investigations |
| EPI1276981 | HA | A/Norway/3221/2018 | 24 Jul 2018 | Norway / Buskerud | Norwegian Institute of Public Health | Bragstad, K; Dudman, SG; Waalen, K; Hungnes, O | Drammen Hospital / Vestreviken HF, Department for Medical Microbiology section Drammen |
| EPI319527 | HA | A/St. Petersburg/27/2011 | 14 Feb 2011 | Russian Federation | National Institute for Medical Research |  | WHO National Influenza Centre Russian Federation |
| EPI390473 | HA | A/Hong Kong/5659/2012 | 21 May 2012 | Hong Kong (SAR) | National Institute for Medical Research |  | Government Virus Unit |
| EPI466626 | HA | A/South Africa/3626/2013 | 06 Jun 2013 | South Africa | National Institute for Medical Research |  | Sandringham, National Institute for Communicable D |
| EPI685579 | HA | A/Michigan/45/2015 | 07 Sep 2015 | United States | Centers for Disease Control and Prevention |  | Michigan Department of Community Health |
| EPI685576 | PB2 | A/Michigan/45/2015 | 07 Sep 2015 | United States | Centers for Disease Control and Prevention |  | Michigan Department of Community Health |
| EPI685577 | PB1 | A/Michigan/45/2015 | 07 Sep 2015 | United States | Centers for Disease Control and Prevention |  | Michigan Department of Community Health |
| EPI685575 | PA | A/Michigan/45/2015 | 07 Sep 2015 | United States | Centers for Disease Control and Prevention |  | Michigan Department of Community Health |
| EPI685572 | NP | A/Michigan/45/2015 | 07 Sep 2015 | United States | Centers for Disease Control and Prevention |  | Michigan Department of Community Health |
| EPI685574 | MP | A/Michigan/45/2015 | 07 Sep 2015 | United States | Centers for Disease Control and Prevention |  | Michigan Department of Community Health |
| EPI685573 | NS | A/Michigan/45/2015 | 07 Sep 2015 | United States | Centers for Disease Control and Prevention |  | Michigan Department of Community Health |
| EPI176620 | HA | A/California/07/2009 | 09 Apr 2009 | North America | Centers for Disease Control and Prevention |  | Naval Health Research Center |
| EPI177327 | PB2 | A/California/07/2009 | 09 Apr 2009 | North America | Centers for Disease Control and Prevention |  | Naval Health Research Center |
| EPI183116 | PB1 | A/California/07/2009 | 09 Apr 2009 | North America | Centers for Disease Control and Prevention |  | Naval Health Research Center |
| EPI176621 | PA | A/California/07/2009 | 09 Apr 2009 | North America | Centers for Disease Control and Prevention |  | Naval Health Research Center |
| EPI176624 | NP | A/California/07/2009 | 09 Apr 2009 | North America | Centers for Disease Control and Prevention |  | Naval Health Research Center |
| EPI176623 | MP | A/California/07/2009 | 09 Apr 2009 | North America | Centers for Disease Control and Prevention |  | Naval Health Research Center |
| EPI176622 | NS | A/California/07/2009 | 09 Apr 2009 | North America | Centers for Disease Control and Prevention |  | Naval Health Research Center |

Table 3. Influenza A(H3N2) viruses WHO Collaboration Centre reference set for 2018/19 season.

| **Segment ID** | **Segment** | **Isolate Name** | **Collection Date** | **Country** | **Submitting_Lab** | **Authors** | **Originating_Lab** |
| --- | --- | --- | --- | --- | --- | --- | --- |
| EPI1255199 | NA | A/Picardie/1688/2018 | 04 Apr 2018 | France | Crick Worldwide Influenza Centre |  | Institut Pasteur |
| EPI1255165 | NA | A/Dijon/1771/2018 | 10 Apr 2018 | France | Crick Worldwide Influenza Centre |  | Institut Pasteur |
| EPI1255155 | NA | A/Alsace/1746/2018 | 05 Apr 2018 | France | Crick Worldwide Influenza Centre |  | Institut Pasteur |
| EPI1145233 | NA | A/Bretagne/1565/2017 | 27 Nov 2017 | France | Crick Worldwide Influenza Centre |  | Institut Pasteur |
| EPI1252525 | NA | A/England/538/2018 | 26 Feb 2018 | United Kingdom | Crick Worldwide Influenza Centre |  | Microbiology Services Colindale, Public Health England |
| EPI1252566 | NA | A/Norway/2620/2018 | 16 Apr 2018 | Norway | Crick Worldwide Influenza Centre |  | WHO National Influenza Centre |
| EPI1252564 | NA | A/Norway/2618/2018 | 16 Apr 2018 | Norway | Crick Worldwide Influenza Centre |  | WHO National Influenza Centre |
| EPI868820 | NA | A/Norway/4465/2016 | 07 Nov 2016 | Norway | Crick Worldwide Influenza Centre |  | WHO National Influenza Centre |
| EPI1274877 | NA | A/Mauritius/2263/2018 | 02 May 2018 | Mauritius | Crick Worldwide Influenza Centre |  | Central Health Laboratory |
| EPI1270813 | NA | A/Mauritius/2287/2018 | 10 May 2018 | Mauritius | Crick Worldwide Influenza Centre |  | Central Health Laboratory |
| EPI1270785 | NA | A/Finland/921/2018 | 16 Apr 2018 | Finland | Crick Worldwide Influenza Centre |  | National Institute for Health and Welfare |
| EPI1270791 | NA | A/Hong Kong/1100/2018 | 12 Jun 2018 | Hong Kong (SAR) | Crick Worldwide Influenza Centre |  | Government Virus Unit |
| EPI1310137 | NA | A/SouthAfrica/R09652/2018 | 27 Jun 2018 | South Africa | Crick Worldwide Influenza Centre |  | Sandringham, National Institute for Communicable D |
| EPI1252576 | NA | A/Tanger/1449/2018 | 20 Mar 2018 | Morocco | Crick Worldwide Influenza Centre |  | Institut National d'Hygi&egrave;ne |
| EPI967220 | NA | A/Greece/4/2017 | 02 Jan 2017 | Greece | Crick Worldwide Influenza Centre |  | Aristotelian University of Thessaloniki |
| EPI1260053 | NA | A/Iceland/78/2018(8925) | 12 Apr 2018 | Iceland | Crick Worldwide Influenza Centre |  | Landspitali - University Hospital |
| EPI1154885 | NA | A/Valladolid/182/2017 | 24 Oct 2017 | Spain | Crick Worldwide Influenza Centre |  | Universidad de Valladolid |
| EPI1255213 | NA | A/Switzerland/8327/2018 | 03 Apr 2018 | Switzerland | Crick Worldwide Influenza Centre |  | Hopital Cantonal Universitaire de Geneves |
| EPI1201231 | NA | A/Switzerland/8060/2017 | 21 Dec 2017 | Switzerland | Crick Worldwide Influenza Centre |  | Hopital Cantonal Universitaire de Geneves |
| EPI1277125 | NA | A/Kazakhstan/A-09/2018 | 04 Apr 2018 | Kazakhstan | Crick Worldwide Influenza Centre |  | National Reference Laboratory |
| EPI1270821 | NA | A/Moscow/186/2018 | 02 May 2018 | Russian Federation | Crick Worldwide Influenza Centre |  | Ivanovsky Research Institute of Virology RAMS |
| EPI781597 | NA | A/Cote D'Ivoire/544/2016 | 06 Apr 2016 | Cote d'Ivoire | Crick Worldwide Influenza Centre |  | Pasteur Institut of Côte d'Ivoire |
| EPI1256087 | NA | A/LaRioja/2202/2018 | 02 Apr 2018 | Spain | Crick Worldwide Influenza Centre |  | Instituto de Salud Carlos III |
| EPI1243179 | NA | A/Bayern/47/2018 | 06 Apr 2018 | Germany | Crick Worldwide Influenza Centre |  | Robert Koch Institute Nationales Referenzzentrum für Influenza |
| EPI1277103 | NA | A/Denmark/795/2018 | 16 Apr 2018 | Denmark | Crick Worldwide Influenza Centre |  | Statens Serum Institute |
| EPI530678 | NA | A/Stockholm/6/2014 | 06 Feb 2014 | Sweden | National Institute for Medical Research |  | Public Health Agency of Sweden |
| EPI539807 | NA | A/Hong Kong/5738/2014 | 30 Apr 2014 | Hong Kong (SAR) | National Institute for Medical Research |  | Government Virus Unit |
| EPI539577 | NA | A/Hong Kong/4801/2014 | 26 Feb 2014 | Hong Kong (SAR) | National Institute for Medical Research |  | Government Virus Unit |
| EPI1047603 | NA | A/Singapore/INFIMH-16-0019/2016 | 14 Jun 2016 | Singapore | Centers for Disease Control and Prevention |  | WHO Collaborating Centre for Reference and Research on Influenza |
| EPI391246 | NA | A/Texas/50/2012 | 15 Apr 2012 | Texas | Centers for Disease Control and Prevention |  | Texas Department of State Health Services-Laboratory Services |
